# The efficacy of wastewater treatment plant on removal of perfluoroalkyl substances and their impacts on the coastal environment of False Bay, South Africa

**DOI:** 10.1007/s11356-024-35509-7

**Published:** 2024-11-18

**Authors:** Cecilia Y. Ojemaye, Adeola Abegunde, Lesley Green, Leslie Petrik

**Affiliations:** 1 Environmental and Nano Science Group, Department of Chemistry, University of the Western Cape, Cape Town, South Africa; 2 Environmental Humanities South and Department of Anthropology, University of Cape Town, Cape Town, South Africa

**Keywords:** Per- and polyfluoroalkyl substances, Seawater, Marine invertebrates, Wastewater treatment plant, Risk assessment, Marine environment, Dispersal zone

## Abstract

Per- and polyfluoroalkyl substances (PFASs), which have their origins in both industrial processes and consumer products, can be detected at all treatment stages in wastewater treatment plants (WWTPs). Quantifying the emissions of PFAS from WWTPs into the marine environment is crucial because of their potential impacts on receiving aquatic ecosystems. In this study, the levels of five PFAS were measured in both influent and effluent sewage water samples obtained from a municipal WWTP, the discharges of which flow into False Bay, on the Indian Ocean coast of Cape Town, South Africa. Additionally, seawater, sediment, and biota samples from eight sites along the False Bay coast were also analysed. Results showed high prevalence of PFAS in the different environmental matrices. Perfluorononanoic acid was most dominant in all these matrices with maximum concentration in wastewater, 10.50 ng/L; seawater, 18.76 ng/L; marine sediment, 239.65 ng/g dry weight (dw); invertebrates, 0.72–2.45 µg/g dw; seaweed, 0.36–2.01 µg/g dw. The study used the chemical fingerprint of five PFASs detected in WWTP effluents to track their dispersion across a large, previously pristine marine environment and examined how each chemical accumulated in different marine organisms. The study also demonstrates that primary and secondary wastewater treatment processes cannot fully remove such compounds. There is thus a need to improve effluent quality before its release into the environment and promote continuous monitoring focusing on the sources of PFAS, including their potential transformation products, their environmental fate and ecological risks, particularly in areas receiving effluents from WWTP.

**Supplementary Information:**

The online version contains supplementary material available at 10.1007/s11356-024-35509-7.

## Introduction

Per- and polyfluoroalkyl substances (PFAS) are known for their chemical and thermal resistance; their amphiphilic and lipophilic properties and their high surface activities (von der Trenck et al. 2018; Verduzco and Wong 2020). They have been broadly manufactured and used as surfactants; firefighting foams; lubricants and polishes and coatings for water resistance products. They are present in consumer goods such as non-stick pots, packaging material for food and leather, furniture, paper and clothing (Sun et al. 2018; Ateia et al. 2019; Ding et al. 2020). The wide range of uses for PFAS has resulted in their widespread presence in the environment (Clara et al. 2008), including the air (Ge et al. 2017), in wild life (Rigét et al. 2013; Surma et al. 2015; Jouanneau et al. 2020; Vendl et al. 2021), oceans and marine biota (Petrik et al. 2017; Ojemaye and Petrik 2019; Vi et al. 2022) and rivers and lakes (Fair et al. 2019; Junttila et al. 2019; Pulster et al. 2022), and they are now found in the Arctic and Antarctic (Macinnis et al. 2017; Garnett et al. 2021).

Extensive toxicological and epidemiological studies have provided evidence linking exposure to PFAS with various health risks including disruption of thyroid functions (Ren et al. 2015), immune suppression (Corsini et al. 2014), irregularity of menstrual cycles (Zhou et al. 2017), inhibition in the activities of steroidogenic enzymes (Chaparro-Ortega et al. 2018; Wang et al. 2018) and impacts on spermatogenesis (Louis et al. 2015; Marcu et al. 2023). These compounds are also neurotoxic (Viberg et al. 2013) and have effects on development, growth and endocrine function (Flynn et al. 2019), affecting the development of foetuses and children, reducing fertility, causing cardiovascular diseases, interfering with the natural hormonal balance within the body, raising cholesterol levels and increasing the risk of cancer (Lind et al. 2017; Starling et al. 2017; Olsen et al. 2017; Osorio-Yáñez et al. 2021). Considering their persistent nature, toxicity, high bioaccumulative potential and ubiquitous distribution (Ikkere et al. 2018), the continual monitoring and eradication of PFAS is important. Currently, the European Chemicals Agency is considering banning all per- and polyfluoroalkyl substances (PFAS) as a class of compounds, due to their extreme persistence, which has caused extensive pollution of the environment and proven accumulation in and harm to humans and other living species (Burger 2023; ECHA 2023).

A key link between communities and the environment is the WWTPs, because the types of chemicals that are being used in communities are reflected in the effluents discharged from these plants. Thus, WWTPs play a significant role in the discharge of PFAS originating from both domestic usage and industrial processes, into the aquatic milieu, particularly those involved in fluorochemical manufacturing or applications. Other sources include atmospheric deposition as well as sites contaminated with firefighting foams (Ahrens and Bundschuh 2014; Liu et al. 2017; Owens 2021). Aquatic organisms face a heightened risk of exposure to PFASs due to the compounds’ high-water solubility, which frequently leads to their presence in these systems. In addition, exposure to a wide range of PFASs can occur through contaminated air, dietary intake and aquatic pathways.

For this reason, perfluorooctane sulfonic acid (PFOS), its salt and perfluorooctanesulfonyl fluoride (PFOSF) were among the persistent organic pollutants (POPs) listed at the Stockholm Convention in May 2009 Annex 2, under restriction (Stockholm Convention 2009). Short-chain perfluorocarboxylic acids (PFCAs) and perfluoroalkane sulphonates (PFSAs) are currently found at increasing levels due to the fact that their precursors are employed as substitutes for PFOS and perfluorooctanoic acid (PFOA). Most PFAS are ubiquitously present in nature or break down into even more persistent metabolites (Wang et al. 2013; Guo et al. 2020; Ojemaye et al. 2023). However, short-chain PFAS (C4-C6) are not without their own negative impacts, although the research on these compounds is still emerging. They are also difficult to eliminate from contaminated sources, as they exhibit the same resistance, physiochemical properties and toxicity, making them a matter of importance (Shi et al. 2017; Xiao 2017; Brendel et al. 2018; Ojemaye et al. 2023), and there is still a paucity of data on their health effect (Sun et al. 2016). South Africa does not produce PFAS and relies on imports, which have been increasing in recent years (Groffen et al. 2018; Batayi et al. 2021), with the chemicals being used extensively in the manufacturing of synthetic textiles; paper and food packaging materials; leather goods, carpets and clothing items and paint, coatings and varnishes. It is therefore likely that WWTPs in the region may contain these chemicals, as confirmed by Adeleye (2016).

False Bay is a geographical expanse located within the Atlantic Ocean, specifically in the vicinity of the Cape Peninsula in South Africa’s Western Cape province. Recreational activities and commercial fishing are the most important economic activities in the bay. Popular recreational activities include swimming, surfing, sailing, scuba and free diving (Pfaff et al. 2019). The increasing population has elevated human-induced impacts on the bay as a result of interference in the form of ecotourism, sand mining, off-road vehicles, bait collecting and trampling along the False Bay shoreline. Other problems include eutrophication, introduction of non-native plant and animal species, alteration of natural habitats, excessive exploitation of marine biodiversity and sewage pollution. (Theron et al. 1992; Compton 2004).

The purpose of this study is to track the chemical fingerprint of sewage effluent using persistent PFASs as tracers so as to determine the extent of effluent dispersal and the contaminant impact zone as well as the environmental consequences upon the marine ecosystem near the shoreline of False Bay. The study also aims to evaluate the accumulation levels of perfluorinated contaminants in biota as a means to gauge potential risks. Considering the limited research on PFAS in False Bay marine environment, this study is pioneering an assessment of PFAS within this marine ecosystem and, by extension, the broader Western Cape Province of South Africa. The primary aim of this article is to fill the knowledge gap regarding PFAS in South Africa’s marine environments. While previous research has focused on freshwater ecosystems and drinking water, this study expands to marine ecosystems, crucial for the region’s ecology and human activities. Our research provides insights into the prevalence, distribution and effects of PFAS in these marine environments, contributing essential data to inform environmental protection, public health and conservation efforts in South Africa.

## Materials and methods

### Sampling sites

**Fig. 1:**
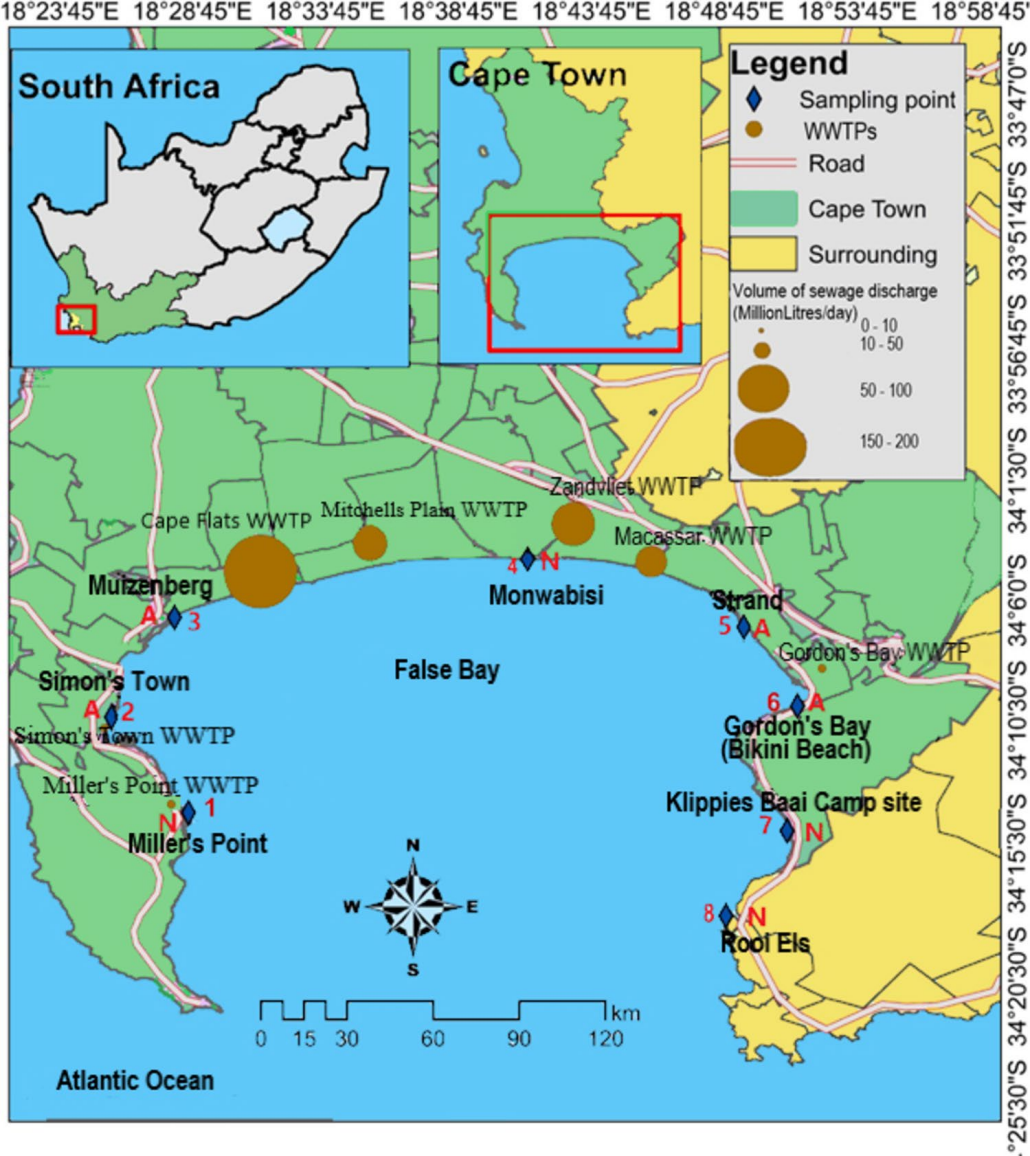
Location of sampling sites and WWTPs in False Bay

False Bay (34°13.19′S 18°38.4′E) is a water body with its widest point being 30 km wide. It is in Cape Town at the south-west end of South Africa where it is warmed by Indian Ocean currents. The Bay is encompassed by Cape Hangklip in the east and the Cape Peninsula in the west. Sampling from eight different sites took place along the False Bay coast (Fig. 1), namely:

- Site 1: Millers Point (34°13′57.9″S 18°28′36.5″E)
- Site 2: Simon’s Town (34°10′22.87″S 18°25′42.73″E)
- Site 3: Muizenberg (34° 6′38.12″S 18°28′5.09″E)
- Site 4: Monwabisi beach (34°04′26.8″S 18°41′21.5″E)
- Site 5: Strand (34° 7′3.71″S, 18°49′29.38″E)
- Site 6: Gordon’s bay/Bikini beach (34°9′57.55″S, 18°51′30.39″E)
- Site 7: Klippies Baai camp site (34°14′39.21″S, 18°51′6.77″E)
- Site 8: Rooi Els ( 34°17′50.65″S 18°48′49.16″E) (Ojemaye and Petrik 2022)

The studied sites were chosen due to their proximity to or distance from WWTP effluent discharges into surf zones or estuaries. False Bay receives effluent from seven different WWTPs that discharge their effluents into False Bay via the surf zone or estuarine discharges, namely Cape Flats WWTP (200 million litres per day (MLD)), Millers Point WWTP (0.06 MLD), Mitchells Plain WWTP (35 MLD), Macassar WWTP (30.7 MLD) Simon’s Town WWTP (1.8 MLD), Gordon’s Bay WWTP (3.4 MLD) and Zandvliet (73.6 MLD) (COCT 2018). During 2017/2018, when sampling occurred, there was a major drought in the Western Cape; thus, very little storm water reached the bay, providing optimal conditions in which to assess the impact of WWTPs as there were low levels of surface run-off. The prevailing current patterns and wind conditions in False Bay exhibit distinct characteristics throughout the year. The surface circulation within the bay generally moves clockwise, influenced by the prevailing southerly winds with cyclonic shearing. In the vicinity of the bay’s entrance, surface currents typically flow westward and are influenced by various factors, including weather patterns, shelf waves, warm Agulhas rings and tidal forces. During winter, when the north-easterly winds dominate, relatively weaker flows are observed in False Bay. Surface currents may display an anticlockwise movement while deeper waters experience cyclonic circulation. However, in the north-eastern corner of the bay, specifically Gordon’s Bay, a recirculation pattern develops, which tends to be cyclonic during north-westerly wind conditions. In contrast, summer brings southeasterly winds that result in a predominantly clockwise circulation at both the epipelagic zone and abyssal zone in False Bay. Along the eastern shore, there is a narrow and robust flow. Nevertheless, in Gordon’s Bay, located in the north-eastern corner of the bay, a counter clockwise gyre is formed (Pfaff et al. 2019). These wind patterns significantly influence the transport of sediment and other particulates within False Bay. Bordering the bay’s western coastline, sediment and particles undergo a northward movement, subsequently veering eastward along the northern coast. Conversely, sediment transport along the eastern shores predominantly occurs in a northward direction (COCT 2019; Pfaff et al. 2019).

In the present study, Zandvliet WWTP (34°03′08.4″S 18°43′05.1″E) was investigated. A privately run WWTP, it was built in 1989 and designed to process 73.6 MLD and serve around 400,000 people. However, with the rapid urbanization and population growth, it was over capacity much sooner than expected. Upgrades were delayed by a decade by several legal battles over the award of the tender, with the result that the Kuils River on which it is situated became heavily polluted by sewage a short distance from its discharge point to the sea.

The primary sources of inflow to the plant consist of sewage and wastewater from municipal and commercial entities. Specifically, the inflow originates from the residential areas of Delft, Blackheath, Thembokwezi, Blue Downs, Eerste River, Khayelitsha, De Wijnlanden, Mxolisi Phetani and southern parts of Kuils River (Zutari 2023) as well as Blackheath Industria and the contaminated military industrial site east of Macassar. The Zandvliet WWTP comprises a screening stage with the removal of grit, which is the preliminary treatment stage, followed by an activated sludge process (AS), which is followed by the membrane bioreactor (MBR) treatment process stage and then by the chlorination treatment stage. The resulting effluent from this treatment process is discharged into the Kuils River which flows into False Bay. Samples were collected at different stages at the plant, namely, influent, after AS treatment, after MBR treatment and at effluent discharge point. The description of other WWTPs has been previously reported (Ojemaye and Petrik 2022).

### Collection and handling of samples

#### Wastewater and seawater

Samples of wastewater were collected (flow- proportional sampling) (US EPA 2013) from the Zandvliet WWTP over a span of 4 months. During this period, access to the facility was granted without any limitations or restrictions. Sampling of Zandvliet effluent was done. Seawater samples were collected close to shore from each marked False Bay site (Fig. 1) from 30 cm below the surface of the seawater. All samples of wastewater and seawater were collected in three replicates in polyethylene bottles (2 L). To ensure sample preservation during transportation to the laboratory, ice was employed. Subsequently, the samples underwent filtration utilizing membrane filters (0.45 µm, 47 mm) and were then stored at a temperature of 4 °C within a refrigerator until the next extraction phase. Bottles of Milli-Q water were used as field blanks, which were transported to the sampling site with other sampling containers and were exposed to the sampling field conditions when sampling.

#### Sediment and biota

Sampling of sediment was done using a grab sampler apparatus (maximum depth of 20 cm) and biota samples: limpet (*Cymbula granatina*) (*n* = 20), mussel (*Mytilus galloprovincialis*) (*n* = 30), sea urchin (*Parechinus angulosus*) (*n* = 15), sea snail (*Oxystele tigrina* and *sinensis*) (*n* = 25), starfish (*Marthasterias glacialis*) (*n* = 10) and seaweed (sea lettuce (*Ulva* sp.), hanging wrack (*Bifurcaria brassicaeformis*), Red algae (*Gelidium pristoides*), strap Caulerpa (*Caulerpa filiformis*) and slippery orbits (Aeodes *Orbitosa*)) were collected along the shore at various points each for the indicated sampling sites in False Bay, South Africa as shown in Fig. 1. Samples of seaweeds, invertebrates and sediment were promptly conveyed to the laboratory after being placed in insulated cooler bags with ice to maintain their freshness and integrity.

Before freeze-drying, all biota samples with exoskeletons were carefully extracted from their shells, and units from each site combined, homogenized and then stored at a freezing temperature of − 80 °C. On the other hand, sediment samples were subjected to room temperature air-drying within the laboratory for 72 h.

All invertebrates, sediment and seaweed samples were kept frozen at − 20 °C prior to evaluation. The selection of PFAS investigated in this False Bay study was built upon prior research (Petrik et al. 2017; Ojemaye and Petrik 2019), as well as their recorded presence in regional and local WWTP effluents (Swartz et al. 2018a).

#### Standards and reagents

Perfluoroundecanoate (PFUnDA), perfluorononanoate (PFNA), perfluoroheptanoate (PFHpA), perfluorooctanoate (PFOA), (100 μg/mL in methanol) and perfluorodecanoate (PFDA) were used for this study, supplied by Sigma-Aldrich (Modderfontein, South Africa) as well as the solvent used (methanol, acetonitrile, and acetone). Oasis HLB 6 cc and 200- and 500-mg cartridges were obtained from Waters (Microsep, South Africa). Milli-Q system (Millipore, Badford, MA, USA) was used to purify the Milli-Q water. All stock solutions were meticulously made-up using methanol in polypropylene (PP) tubes/vials and stored at a temperature of 4 °C.

### Preparation of samples

#### Water samples

Wastewater and seawater (500 mL) extractions were done according to Petrik et al. (2017) using Oasis HLB 6 cc of 500 mg SPE cartridges. Briefly, preconditioning of SPE cartridges was done with 7 mL each of methanol and Millipore water. Five hundred millilitres of aqueous sample was adjusted to a pH of 6 and drawn through the cartridges under vacuum. After conditioning of the SPE cartridges, elution of the analytes was achieved using 7 mL of methanol which was thereafter reduced to a volume of 2 mL using a stream of nitrogen. For each sample, internal standards (50 µL of a 50 ng/mL solution) were added. The resulting extracts were centrifuged and then passed through a 0.2-μm syringe filter. Finally, the filtered extracts were transferred to vials for analysis and quantification using liquid chromatography-tandem mass spectrometry (LC–MS/MS).

#### Sediment, seaweeds and invertebrates

Our previously established method (Ojemaye and Petrik 2019) was applied to extract solid samples. Around 10 g of each freeze-dried, combined sample was weighed out exactly and placed into an extraction thimble. The extraction process involved utilizing a methanol/acetone mixture in a ratio of 3:1 (v/v), with 100 mL of the solvent mixture in a Soxhlet extractor. The resulting extract was then evaporated down and reduced to a volume of 10 mL using a rotary evaporator. To precipitate lipids, NaOH (1 M) was used to adjust the pH of the extract to 6. The sample was subsequently subjected to centrifugation at 3000 rpm for 1200 s. The supernatant was decanted into a PP bottle and made up to a final volume of 100 mL with Milli-Q water. The SPE procedure (6 cc, 200 mg cartridge) used for the samples of seawater was also used for the biota extracts and eluted at a flow rate of 1 mL/min.

#### LC-MS/MS analysis

The detailed protocols for LC–MS/MS analysis used were described in previous studies (Petrik et al. 2017; Ojemaye and Petrik 2019). Briefly, PFASs extracted from seawater, wastewater, sediment and biota samples were identified and quantified using Water Acquity UPLC with binary solvent manager and sample manager coupled to a Xevo Triple quad mass spectrometer fitted with an electrospray ionization source. Analytes were detected and quantified using multiple reaction monitoring (MRM) mode. The 1.7-μm ACQUITY UPLC BEH C18 column (Waters, Mildford, MA, USA) with dimension of 2.1 × 100 mm was used for separation at a temperature of 60 °C.

### Quality assurance

The linearity, limit of detection (LOD), limit of quantification (LOQ), precision and the selectivity and accuracy of the analytical procedure were evaluated and determined. Evaluation of selectivity was based on qualitative identification of the compound of interest, by comparing the sample’s peak spectra and retention time to what was obtained for the standard. The evaluation of precision involved the calculation of the relative standard deviation (RSD) from analysing five replicate injections of both the selected standard and sample. The obtained RSD values were below 15%. The method’s linearity was evaluated using the linear correlation coefficient (*R*^2^) method. Eleven-point calibration curves were constructed within a concentration range for each standard of 0.1–1000 ng/L. The resulting *R*^2^ values obtained from the analysis were greater than 0.995, indicating a strong linear correlation. To assess the accuracy, percentage recoveries were calculated through comparison of the concentrations in pre-spiked samples and those in post-spiked samples. The results of the recovery analysis for each analyte can be found in Table 1.

**Table 1:** Quality parameters of all analysed compounds

Compound name	MW (g/mol)	RT (min)	Log kow	Ion transition (m/z)	CE (eV)	LOD				LOQ				Recovery (%)			
						Seawater (ng/L)	Wastewater(µg/L)	Biota (ng/g)	Sediment (ng/g)	Seawater (ng/L)	Wastewater (µg/L)	Biota (ng/g)	Sediment (ng/g)	Seawater	Wastewater	Biota	Sediment
PFHpA	364.06	7.00	6.86	363 to 319	15	0.01	0.03	0.38	0.38	0.03	0.08	1.15	1.15	100.9	90.0	98.3	96.7
PFOA	414.07	7.56	7.75	413 to 369	15	0.02	0.02	0.45	0.45	0.07	0.08	1.37	1.37	99.9	98.3	98.9	99.7
PFNA	464.08	8.03	8.64	463 to 419	15	0.01	0.02	0.81	0.81	0.02	0.06	2.46	2.46	101.2	100.0	99.0	100.8
PFDA	514.09	8.41	9.53	513 to 469	15	0.002	0.02	0.45	0.45	0.01	0.69	1.37	1.37	100.7	99.0	98.2	99.4
PFUnDA	564.09	8.74	10.42	563 to 523	15	0.002	0.04	0.47	0.47	0.01	0.11	1.42	1.42	99.6	90.5	98.9	99.8

*MV* molecular weight, *CE* collision energy, *RT* retention time, *LOD* limit of detection, *LOQ* limit of quantification

LOD and LOQ were calculated for each analyte using 3.3 * *σ*/*S* and 10 * *σ*/*S* respectively where *σ* = standard deviation (SD) of blank-sample responses and *S* = slope of the calibration curve. Prior to use, all equipment, glassware and sampling bottles were rinsed with methanol and water so as to avoid analytical interference and cross contamination. To account for possible contamination in both the laboratory and field sampling, field and procedural blanks were conducted as control measures. Blank samples were included and analysed alongside the regular samples. For the studied compounds, no contaminations of PFAS were found above the detection limit in blanks.

### Statistical analysis

Statistical Package for Social Sciences (SPSS) was applied for statistical analysis IBM version 20 software. The replicate data were expressed as mean ± standard deviation (SD) and were analysed using the Duncan New Multiple Range Test for separation.

### Mass load for PFAS

The mass load of PFAS in wastewater samples was calculated according to Eqs. (1) and (2):

1 $$\text{Mass load }\left(\text{mg}/\text{d}\right)=C \times Q$$

2 $$Q ={Q}_{\text{in}} -{Q}_{\text{PS}} -{Q}_{\text{WAS}}$$

Where:

- *C* = compound’s concentration in effluent, in mg/ML
- *Q* = effluent flow rate (ML/d)
- *Q*_PS_ = primary sludge flow rate
- *Q*_WAS_ = flow rate waste activated sludge
- *Q*_in_ = flow rate of influent
- The calculation of *Q* yielded a value of 60.88 ML/d, derived from an average influent flow rate of 84.38 ML/d, with no PST sludge wasting rate (0.00 ML/d), and an activated sludge wasting rate of 0.302 ML/d.

### Environmental risk assessment (ERA)

The risk quotient (RQ) was determined by calculating the ratio between the point estimate of exposure and the point estimate of effects, following the methodology outlined by the US Environmental Protection Agency (EPA) (US EPA 2016). The RQ was calculated for each analyte detected in the seawater collected from the oceanic surroundings of False Bay, using a worst-case scenario.

#### Procedure to calculating the cumulative environmental risk assessment

In order to assess the cumulative risk, we followed the tiered method advocated by Backhaus and Faust (2012). The first tier involves calculation of the risk quotient (RQ_(MEC)_) of a mixture of known composition summing up the MEC/PNEC ratio of the components, where MEC is equal to the measured environmental concentration, and PNEC is equal to the predicted no-effect concentration. In order to estimate the worst-case scenario during the observed period, the maximum measured concentrations of the target compound in seawater were utilized for calculating the RQ. This evaluation was estimated based on the toxicity data found in literature for algae, invertebrate and fish, as provided in SI Table 1 with the corresponding assayed species, endpoint, including the assessment factor (AF) (SI Table 2) as advised by the European Chemical Agency (ECB 2003).

3 $${\text{RQ}}_{(\text{PNEC})}=\sum \nolimits_{i=1}^{n}\frac{{MEC}_{i}}{{PNEC}_{i}}=\sum \nolimits_{i=1}^{n}\frac{{MEC}_{i}}{min({EC50}_{\text{algae}};{EC50}_{\text{invertebrates}};{EC50}_{\text{fish}})}\times {AF}_{i}$$

This formula does not adhere to the concentration addition (CA) principle because the PNEC values are based on toxicological data obtained from different organisms and, in the RQ calculation, are therefore summed up as ratios that consider species belonging to various trophic levels. However, since we chose the lowest toxicological values, this approach follows the precautionary principle, and it is possible to use it for a preliminary ERA.

If RQ_(PNEC)_ is above 1, potential risk cannot be excluded, and the calculation needs to be refined by using a second approach which is based on the toxic units (TUs). TUs are determined by calculating the ratios between the concentration of a specific compound (*i*) in a mixture and the concentration of that same substance that provokes *x%* effect if present alone (here we consider 50%):

4 $$\text{TU}= \frac{{MEC}_{i}}{{EC50}_{i}}$$

The TUs are calculated for each group of organisms, and, for each group, the TUs of all contaminants are summed up in the sum of toxic unit (STU). In total, 3 STUs are calculated (Eq. 5):

5 $$\text{STU}=\sum \nolimits_{i=1}^{n}{TU}_{i}= \sum \nolimits_{i=1}^{n}\frac{{MEC}_{i}}{{EC50}_{i}}$$

A single RQ value—follow as RQ_(STU)_—is calculated considering the STU of the extremely sensitive trophic level and an AF = 1000:

$${\text{RQ}}_{\left(\text{STU}\right)}=max\left({STU}_\text{algae};\;{STU}_\text{invertebrates};\;{STU}_\text{fish}\right)\times AF=$$

$$=\mathit{max}(\sum \nolimits_{i=1}^{n}\frac{{MEC}_{i}}{{EC50}_{i, \text{algae}}} ,\sum \nolimits_{i=1}^{n}\frac{{MEC}_{i}}{{EC50}_{i, \text{invertebrates}}} , \sum \nolimits_{i=1}^{n}\frac{{MEC}_{i }}{{EC50}_{i, \text{fish}}})\times AF$$

## Results and discussion

All samples of seawater, sediment, seaweeds and invertebrate species were collected from eight distinct coastal locations within the oceanic surroundings of False Bay depicted in Fig. 1. Table 1 presents the quality parameters of LC–MS, including percentage recoveries, LOD and LOQ for the analysis of each identified PFAS contaminant in the different matrices evaluated in this study.

### Wastewater

The wastewater samples collected in triplicate at different treatment stages from Zandvliet WWTP are presented in Table 2. The WWTP is an activated sludge plant type with no tertiary treatment process apart from chlorination.

**Table 2:** The mean concentration (in ng/L) and corresponding standard deviation and mass load (mg/d) of PFAS in the samples of wastewater from Zandvliet WWTP. *AS* activated sludge, *MBR* membrane bioreactor, *LOQ* limit of quantification, *LOD* limit of detection

Concentration					Mass load	
Treatment	Influent	AS	MBR	Final effluent	Influent	Final effluent
PFHpA	48.53	32.32(0.1)	< LOD	34.51	4094.96	2100.97
PFOA	13.79	7.53(0.1)	5.62(0.1)	16.71	1163.6	1017.31
PFNA	22.35	10.10(0.2)	10.50(0.1)	10.03	1885.89	610.63
PFDA	4.3	< LOD	< LOD	3.86	362.83	235.0
PFUnDA	< LOQ	< LOD	< LOD	10.12	0	616.11
ΣPFCs	88.97			85.26	7507.29	4580.00

Table 2 shows the sum of the average concentration of PFAS in the influent was 88.97 ng/L, while in the effluent, it was found to be 85.26 ng/L. Among the PFASs, the highest recorded concentration was 48.53 ng/L for PFHpA, observed in the influent samples. PFNA was the prevailing PFAS among all the five evaluated PFASs, followed by PFOA, PFHpA and PFDA which were all detected in the influent of the WWTP while PFDA and PFUnDA were the only compounds not quantified in the activated sludge of the WWTPs. PFHpA had the highest concentration in the effluent. The dominancy of PFNA may reflect the current use in this region. Consistent with the findings of Arvaniti et al. (2012), Coggan et al. (2019) and Sun et al. (2011), similar levels of PFAS were observed in different stages of treatment in this study. However, the detected PFAS levels were lower compared to the effluent of WWTPs in certain other countries. For instance, significantly higher levels of PFAS ranging from 10 to 635 ng/L were reported in a WWTP located in the Kanto area of Japan (Murakami et al. 2009) and Beijing, China (12.9–691.8 ng/L) (Gu et al. 2021), while comparable levels were found in studies from Sweden (18–66 ng/L) (Eriksson et al. 2017) and China (0.01–107.0 ng/L) (Zhang et al. 2013). In different regions, lower concentrations of PFOA and PFNA have been detected. For example, in North Spain, concentrations ranged from 0.16 to 3.53 ng/L for PFOA and from 0.06 to 1.40 ng/L for PFNA, as reported by Gómez et al. (2011). Similarly, in Southeast China, concentrations ranged from 0.025 to 5.20 ng/L for PFOA and PFNA (Zhang et al. 2016).

Additionally, previous studies have demonstrated that PFOA exhibits non-biodegradability during the activated sludge process, as noted by Sinclair and Kannan (2006). Moreover, there was a noted rise in PFOA concentrations in the effluent of Zandvliet WWTP. Similar trends were published by Kunacheva et al. (2011), Chularueangaksorn et al. (2012), Loganathan et al. (2007) and Sinclair and Kannan (2006).

It is evident that momentary fluctuations of compounds occur in the aqueous phase when entering and exiting the WWTP. It is impossible to assert that the influent portion sampled is the same portion sampled at the exit because of the flow speed and residence time of a particular increment of influent as it passes through unit operations. Thus, it is likely that effluent concentrations may be higher or lower and removal cannot be solely ascribed to unit operations but also to fluctuation in influent concentrations. According to some authors, the reason for the increase in the concentration of PFAS in the effluent prior to the discharge is due to the contaminants adsorbed on the sludge being released into the effluent (Loganathan et al. 2007; D’eon 2010; Eriksson et al. 2017; Gallen et al. 2018; Kim Lazcano et al. 2019). For example, a study conducted by Loganathan et al. (2007) examined the concentrations of PFASs in water treatment systems and found levels ranging from 1.4 to 83 ng/L in the influent and from 2.2 to 155 ng/L in the effluent. This study indicates that the concentration of PFAS tends to increase through the treatment process, leading to higher levels in the effluent compared to the influent. Similarly, research by Gallen et al. (2018) reported PFAS concentrations between 0.98 and 440 ng/L in the influent and between 21 and 560 ng/L in the effluent. Both studies consistently show that PFAS levels are elevated in the effluent, highlighting the persistence and potential accumulation of these substances through water treatment processes. Precursors of some PFAS like fluorotelomer alcohols, polyfluoroalkyl phosphate esters and sulphonamides may degrade during the treatment process in the treatment plant, which subsequently could contribute to the presence of PFOA (Eriksson et al. 2017; Winchell et al. 2021). The results show the lack of effective removal mechanisms for PFAS such as PFOA during the primary and secondary treatment processes, as other authors report as well (Kunacheva et al. 2011; Chularueangaksorn et al. 2012; Chiavola et al. 2020).

The input concentrations and types of PFAS in sewage can change prior to wastewater treatment, and this affects its distribution downstream in different environment matrices. The findings of this study relating to PFOA are consistent with the explanations.

The mass loadings of the specific PFAS were approximated for both influent and effluent samples. Table 2 displays the calculated mass loads of PFAS for an individual process unit within the WWTP under study. From Zandvliet WWTP effluent and influent, PFHpA exhibited the highest mass load at 2100.97 and 4094.96 mg/d respectively, while the lowest mass load of 235.0 and 362.83 mg/d was for PFDA respectively. The total mass loads were 7507.09 mg/d for influent and 4580.00 mg/d for effluent.

Since PFASs are among the chemicals of emerging concerns (CECs), currently, there is a paucity of information regarding their guideline limit in treated wastewater. However, in some countries, there are guideline values put in place for some of these PFASs in potable water. In 2016, according to the USEPA, an advisory limit of PFOA is 70 ng/L in drinking water (USEPA 2016) while in 2022, it is 0.004 ng/L (U.S. EPA 2022). The precautional limit for PFOA in Germany for drinking water was set to a tolerable level of 200 ng/L for consumption in a year (GDWC 2017), while in Massachusetts, Michigan, Minnesota, New York and New Jersey, the health risk limits of 20 ng/L, 8 ng/L, 35 ng/L, 10 ng/L and 14 ng/L respectively were set for PFOA (Post 2021). The health-based value for PFOA in drinking water in Canada is 200 ng/L (Health-Canada 2018).

Recently, the EPA has finalized a National Primary Drinking Water Regulation (NPDWR) that sets legally enforceable maximum contaminant levels (MCLs) for six PFAS compounds in drinking water. The MCLs of these compounds are 4.0 ng/L for PFOA and PFOS and 10 ng/L for PFHxS, PFNA and HFPO-DA. Additionally, the regulation addresses PFAS mixtures that contain two or more of PFHxS, PFNA, HFPO-DA and PFBS by using a hazard index MCL to consider the combined and co-occurring levels of these PFAS in drinking water which is set to be 1. The EPA has also established health-based, non-enforceable maximum contaminant level goals (MCLGs) for these substances which are 0 ng/L for PFOA and PFOS and 10 ng/L for PFHxS, PFNA and HFPO-DA (US EPA 2024).

The data in Table 2 indicates that PFASs are present in effluent discharged from Zandvliet WWTP. Moreover, several other WWTPs also release these compounds in their partially treated effluents (Adeleye 2016; Swartz et al. 2018b).

Recent studies have demonstrated that PFAS removal from water/wastewater can be achieved using various techniques. These include adsorption methods involving activated carbon (Park et al. 2020; Niarchos et al. 2022) and nanomaterial (Omo-Okoro et al. 2021; Yin et al. 2023) and filtration techniques such as membrane technologies (Das and Ronen 2022), nanofiltration (McCleaf et al. 2023) and photocatalytic ozonation (Lashuk et al. 2022). Incorporating one or more of these methods into the existing WWTPs could optimize treatment processes and significantly enhance PFAS removal efficiency.

### Seawater

**Fig. 2:**
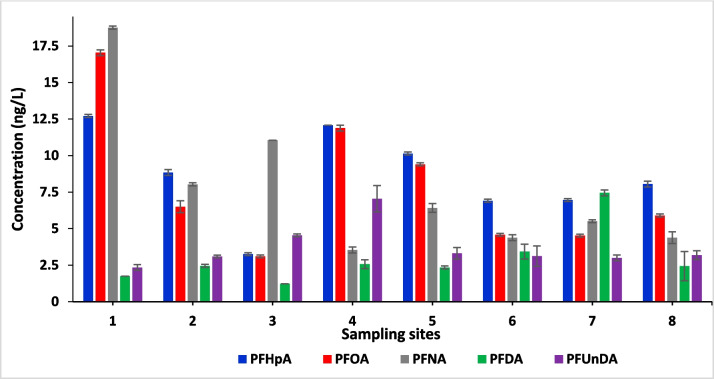
The levels of PFAS in seawater samples collected from various sites across False Bay

Figure 2 presents the levels of the five selected PFAS found in seawater collected at eight sites in False Bay as shown in Fig. 1. These compounds were detected in sewage effluent discharged from the Zandvliet WWTP emptying into False Bay as shown in Table 2.

The levels of PFAS measured in seawater at each site were fairly consistent across the locations and areas with all the monitored PFAS in this study detected across all the sites (Fig. 2). From the results at sampling sites 1 and 3, PFNA concentration was observed to be 18.76 ng/L and 11.05 ng/L respectively, higher than other PFASs while PFDA had the lowest concentration of 1.75 ng/L and 1.23 ng/L at these two sites. Similar trends were found at sampling site 2, 4, 5 and 8, and PFHpA concentration was 8.84 ng/L, 12.08 ng/L, 10.14 ng/L and 8.05 ng/L respectively. The PFAS with the lowest concentration at those sites was PFDA with concentrations of 2.45 ng/L, 2.57 ng/L, 2.34 ng/L and 2.43 ng/L respectively. PFHpA had the highest seawater concentration of 6.91 ng/L and PFUnDA the lowest concentration of 3.43 ng/L in site 6. This observation is different to what was found in other sites. On the other hand, a contrasting observation was presented in seawater samples from sampling site 7; PFDA exhibited the highest concentration of 7.45 ng/L, and PFUnDA had the lowest concentration of 3.00 ng/L.

Overall, considering the consistency of PFAS concentrations across the locations and sites in this study, it was observed that there was a gradual decrease in the concentration of PFAS from sampling site 1 to site 8. However, significant levels of these PFAS were detected at all the sampling sites. Also, of all the sampling sites, sites 1 to 5 presented the highest ∑PFAS which ranged from 23.17 to 52.6 ng/l. The WWTPs (Zandvliet, Cape Flats, Miller’s Point, Macassar, Simon’s Town, Mitchells Plains and Gordon’s Bay) are all pumping partially treated sewage into sea. PFASs are present in effluents discharged from Zandvliet WWTP, and these compounds are also released in effluent from other WWTPs in the Western Cape (Adeleye 2016; Swartz et al. 2018b).

Cape Town Department of Environmental Affairs and Tourism on marine waters (DEAT) states that the city’s coastline is imperilled by several pressures and drivers, from mining activities and coastal development to runoff of storm water and discharge of effluent (Department of Environmental Affairs and Tourism (DEAT) 2005). The levels of the evaluated PFAS in this study relate to the observations of DEAT. Moreover, it is important to highlight that the concentrations of PFAS detected in False Bay were higher in comparison to the concentrations found in samples from other locations (Petrik et al. 2017; Ojemaye et al. 2022a). This difference could be attributed to the volume of sewage discharged into the bay, which is significant, ranging from 0.6 to over 200 million litres per day from seven different WWTPs.

Marine background levels of PFAS, unaffected by sewage treatment plants, are generally low but can vary depending on location. For example, studies conducted in the Norwegian Arctic show low PFAS concentrations in marine environments, with values often used as baseline references for uncontaminated sites (Ali et al. 2021b). In comparing the concentrations of PFAS in this specific study with levels found in other studies, similar findings were observed in water samples obtained from Ría de Vigo in Spain and South Korea, where the concentrations of PFHpA, PFOA, PFDA, PFNA, and PFUnDA ranged from 0.1 to 29 ng/L (Hong et al. 2015). However, the concentrations were higher compared to the present study in water collected from Saudi Arabia’s Red Sea, and Korean east and west coast seawater and Shandong peninsula ranged between < LOQ and 956 ng/L (Naile et al. 2013; Son 2017; Wan et al. 2017; Ali et al. 2021a). Conversely, the levels of PFAS reported in seawater from the Mediterranean Sea (Brumovský et al. 2016), Osaka Bay and coastal waters of Western Japan (Beškoski et al. 2017), coastal seas of Hyogo in Japan and North-western Atlantic Margin (Takemine et al. 2014; Zhang et al. 2019) were lower than the levels found in this study. The highest levels of PFOA (ranged from 13.6 to 441 ng/L) were found in the Bohai Sea, Baiyangdian Lake and Yellow Sea (Zhao et al. 2017; Guo et al. 2020), while the levels found in samples from the Bohai Sea (9.9 ng/L) (Chen et al. 2016) were lower compared to the present study. PFAS detention in wastewater has been reported in this region (Swartz et al. 2018b), due to their use in many consumer goods by humans. Because these PFASs are known not to degrade in the environment, their detection in the seawater in this area could cause their bioaccumulation in marine life.

### Sediment

Sediment samples were taken from the oceanic environment of False Bay, specifically from sites 1 to 8, to study the presence of contaminants. Sediment plays an important role in the transport of contaminants as highlighted by Taleb et al. (2007). It influences the concentration of contaminants in the water column and their accessibility to filter-feeding organisms, thereby influencing their bioavailability. The geographical configuration of False Bay exerts an influence on the dynamics of marine water, as indicated by Taljaard et al. (2000). The influence of the bay’s shape and features, such as its coastlines and underwater topography, can affect the distribution and movement of substances like contaminants in the water. In this case, it suggests that the similarities in the concentrations of contaminants observed throughout the bay may be attributed to the specific way the bay is structured, which affects how these substances disperse and accumulate. This aspect may account for the observed similarities in the concentrations of contaminants across the bay.

**Fig. 3:**
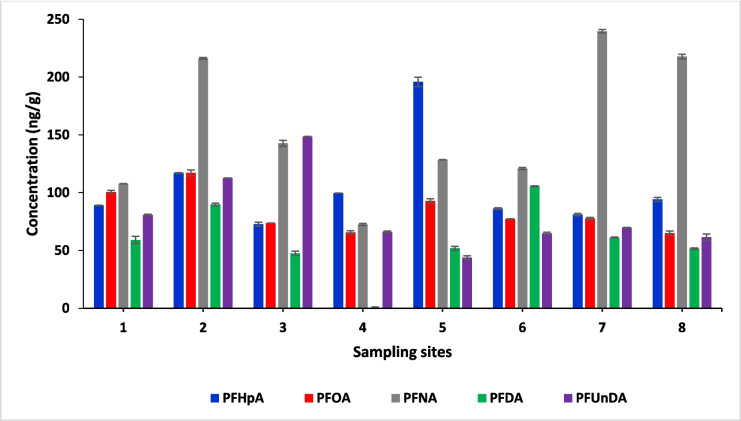
Concentrations found in sediment samples across all locations in False Bay

The result showed that PFAS levels in sediment samples were elevated compared to seawater samples. The accumulation of these chemicals in sediment occurs due to its role as a sink or reservoir. This observation aligns with previous reports on the subject (Shi et al. 2010; Lin et al. 2020). PFASs have a moderate to high adsorption rate on sediment and high solubility (Wang et al. 2019, 2021). All the PFASs investigated were detected in all the sediment samples, and their concentration in dry weight (dw) ranged from PFUnDA, 43.85 to 148.46 ng/g; PFDA, 47.54 to 105.51 ng/g; PFNA, 72.52 to 239.65 ng/g; PFOA, 64.98 to 117.09 ng/g and PFHpA, 72.72 to 195.79 ng/g (Fig. 3). The study found that long-chain PFASs are less soluble and more likely to adsorb onto sediment than short-chain PFASs. The study found that the concentration of PFAS in False Bay sediment was higher than in other locations studied. For instance, PFAS concentrations in sediment analysed from Indonesian coastal water (0.25–6.1 ng/g) (Harino et al. 2012); Bohai Sea, China (0.06–2.98 ng/g dw) (ND–4.3 ng/g dw) and the East China Sea (0.03–1.77 ng/g dw) (Chen et al. 2011; Gao et al. 2014); Shandong peninsula, China (1.30–11.17 ng/g) (Wan et al. 2017); Hong Kong (0.10–1.59 ng/g dw) (Loi et al. 2013); Bengal coast (1.07–8.15 ng/g) (Habibullah-Al-Mamun et al. 2016) and Cantabrian Sea, Spain (0.01–0.13 ng/g) (Gómez et al. 2011). The higher concentration found in this study may be due to increased sewage/wastewater discharge from the surrounding areas as a result of fast population growth resulting in high urban development. The concentrations of PFAS and PFDA found in the sediment from Osaka Bay (31–9500 ng/g) and Camps Bay (150 ng/g) respectively were however higher (Beškoski et al. 2017; Ojemaye et al. 2022b). The reported levels of PFCs in ground and surface water samples have been reported to fall within the range of several hundred micrograms per litre while sediment samples have exhibited concentrations ranging from several hundred micrograms per kilogram to several thousand milligrams per kilogram as reported by Arvaniti et al. (2015). These findings are consistent with the results of this study. The sediment samples collected in False Bay demonstrate a higher concentration of all detected compounds compared to the water column, where the concentrations were only at the nanograms per litre level. In recent times, the City of Cape Town has officially stated that the water quality along its coastline is of low quality with False Bay among the affected areas, which supports the findings of this study (COCT 2019).

#### The distribution coefficients

The concentration of contaminants in sediment and seawater at the same sampling sites was used to estimate the sorption coefficients of the contaminants between these two phases. The sorption coefficient *K*_*d*_, as described by Kozerski et al. (2014), signifies the reversible sorptive interaction of chemicals between sediment and water. It is determined using Eq. (6):

6 $${K}_{d}={~}^{{C}_{\text{ss}}}\!\left/ \!{~}_{{C}_{\text{ws}}}\right.$$

The variables *C*_ss_ and *C*_ws_ denote the *K*_*d*_ of compounds in sediment samples (dw) and water samples respectively.

**Fig. 4:**
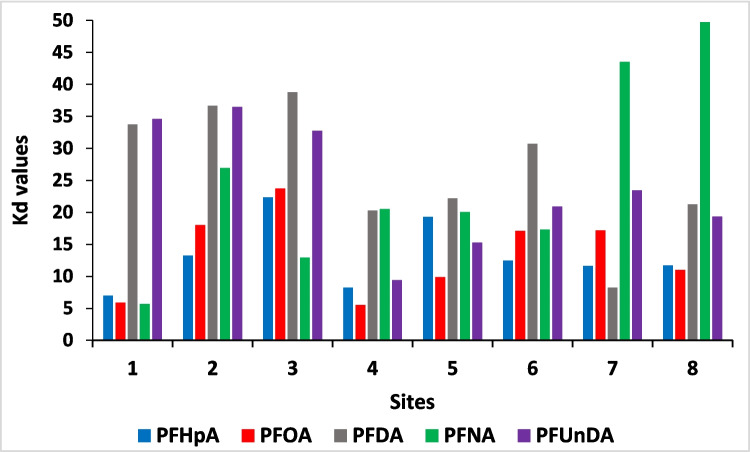
Sediment *K*_*d*_ values for PFAS at various sites around False Bay

Figure 4 displays the *K*_*d*_ values associated with the sampling sites. It should be noted that these values are only approximations as they do not precisely represent equilibrium conditions. This is primarily attributed to the significant variability observed in seawater concentrations as well as site-specific variations. The *K*_*d*_ values for perfluorinated compounds ranged between PFHpA (7.00–22.38 L/kg), PFOA (5.33–23.72 L/kg), PFUnDA (9.40 to 36.47 L/kg), PFNA (5.71–49.72 L/kg) and PFDA (8.22–38.76 L/kg), as determined in this study.

The *K*_*d*_ values of each compounds varied significantly. Generally, the *K*_*d*_ values showed that all the tested compounds did not exhibit strong affinity for sediment with *K*_*d*_ ≤ 50 L/kg in all sites and hence are not sorbed to an appreciable degree unlike the longer chains C > 11 which often exhibit a stronger sorptivity. However, the variation observed for all the compounds from the different sites showed that the movement and dispersion of the compounds through the water, influenced by ocean currents and flow patterns, play a dominant role in causing concentration variation, rather than their tendency to adhere strongly to sediment. It is important to consider other possible processes, such as chemical or biological transformation, when interpreting these data. Such processes may impact the degree of persistence and/or concentration of contaminants in sediments, even over short periods of time. While PFASs are generally persistent, changes in conditions can affect their bioavailability and ecological risks. In complex environmental systems, interactions between various processes can yield unexpected outcomes and behaviours for persistent compounds like PFASs. A *K*_*d*_ value that is very low indicates that contaminants are more mobile in water than in sediments, as discussed by Gouin et al. (2016).

### Marine invertebrates

**Fig. 5:**
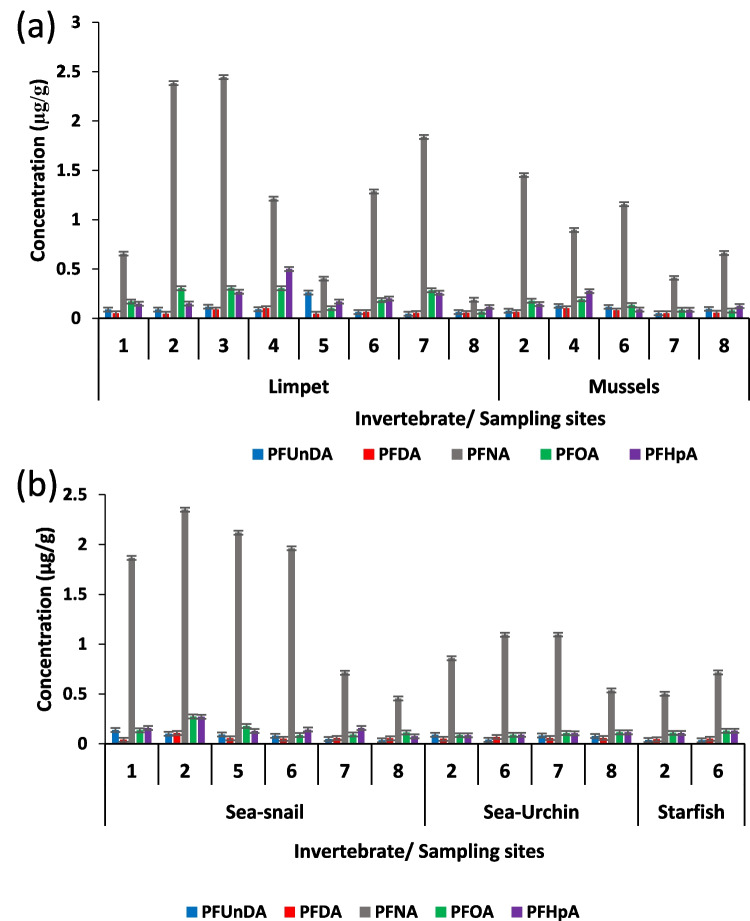
PFAS concentration in invertebrate found in different sites

The concentrations of PFAS in various marine organisms, namely sea urchin, limpet, starfish, sea snail, and mussels, were examined at each of the eight locations within False Bay (Fig. 5). The choice of these organisms was determined by their prevalence and abundance across the different sites within the study area. Given that Taljaard (2006) reported the presence of emerging contaminants in sewage outfalls and wastewater treatment plants that flow into False Bay, conducting an evaluation of pollutant levels in marine organisms from this specific region is important. Typically, the concentrations of numerous contaminants in organisms are notably higher compared to seawater and sediment samples (Ojemaye et al. 2022b). The levels of PFUnDA, PFDA, PFHpA, PFNA and PFOA were examined in five different marine organisms from eight sites in False Bay. This study marks the first evaluation of PFAS in marine organisms from this bay, with all biota samples reported in dry weight (dw) and in microgram per gram (µg/g).

Limpet samples were collected from all eight sites within the study area. The concentrations of PFAS varied within the following range: PFUnDA (0.0463–0.2608 µg/g dw), PFDA (0.0456–0.1019 µg/g dw), PFNA (0.1874–2.4449 µg/g dw), PFOA (0.0650–0.3082 µg/g dw) and PFHpA (0.1158–0.2695 µg/g dw).

The detected levels of PFAS in mussels varied within the following range: PFUnDA (0.0506–0.1258 µg/g dw), PFDA (0.0511–0.1026 µg/g dw), PFNA (0.4100–1.4521 µg/g dw), PFOA (0.0786–0.1922 µg/g dw) and PFHpA (0.0875–0.2756 µg/g dw).

The samples collected from site 4 exhibited the highest concentrations of PFHpA, PFDA, PFOA and PFUnDA among all the sites in this study, while PFNA had the highest concentration at site 2. The pattern of PFAS concentrations in mussels followed a similar trend to that of limpets (PFNA > PFOA > PFHpA > PFUnDA > PFDA).

Sea snail samples varied in the range of 0.0356–0.1378 µg/g dw, 0.0398–0.1091 µg/g dw, 0.4554–2.3504 µg/g dw, 0.0891–0.2745 µg/g dw and 0.0757–0.2709 µg/g dw, respectively.

Sea urchin samples exhibited lower levels of PFAS compared to sea snail, mussels and limpet samples. In starfish samples, PFNA was found to have the highest concentration of 0.5016 and 0.7162 µg/g at sites 2 and 6 respectively, although these levels were lower than what was found in other invertebrate and for other PFC compounds.

The relative abundance of PFUnDA, PFHpA, PFNA and PFOA across the various species followed the order: limpets > sea snail > mussels > sea urchin > starfish. As for PFDA, the order of abundance was sea snail = mussels > limpet > sea urchin > starfish. The observed trend was identified by analysing the prevalence of PFAS in each species across multiple sites. The relative concentrations of PFAS in various invertebrates indicate that the bioaccumulation of these compounds is about a thousand-fold higher than levels found in seawater or sediments. Uptake was species-specific and dependent on the specific PFAS compound within water and biological systems. In addition, the chemical properties of the PFAS, such as lipophilicity (Log *K*_ow_), molecular size and functional groups are properties that affect how PFASs interact with biological membranes and tissues, contributing to the observed accumulation patterns. For example, PFASs with higher lipophilicity (higher Log Kow) are more likely to accumulate in the lipid-rich tissues of organisms, explaining the variations in PFAS concentrations among different species.

Mussels are filter feeders and have the ability to accumulate contaminants from water and food, making them useful as indicator species to monitor the quality of marine environments (Edward 2013; Silva et al. 2017; Krishnakumar et al. 2018). However, limpets have also demonstrated potential as a pollution indicator in this study.

Sea urchins are recognized as a valuable indicator species for assessing local pollution levels, due to their sensitivity and habit of adapting to variations in environmental conditions. Due to their biological and biochemical attributes, they are well-suited for monitoring and assessing the impact of pollution on the marine ecosystem (Bayed et al. 2005; Soualili et al. 2008; Angioni et al. 2012). But in the case of this study, they were not present at each site, which made limpets more useful.

The differences observed in the levels of PFAS among the various invertebrates sampled from different sites may be attributed to physiological factors, including diet preferences, egestion rates, age and growth rates, metabolic capacities and respiration patterns of the organisms (Tomy et al. 2004; Falk et al. 2015). These factors can influence the accumulation and rate of elimination of PFAS in the organisms, leading to variations in their concentrations. The findings suggest that the availability and patterns of exposure to contaminants differ between seawater, sediment and organisms, highlighting variations in the ways organisms process and interact with these substances.

The detection of these compounds in all samples from every site and in the WWTPs indicates the extensive influence of sewage effluents on the marine environment as sewage indicator chemicals like pharmaceuticals have been previously reported in this marine environment (Ojemaye and Petrik 2022). The elevated levels of PFAS detected in all invertebrates can be attributed to the constant discharge of partially treated sewage into surf zones that increase the concentration of contaminants along the shoreline of False Bay, leading to bioaccumulation in marine organisms.

In this study, we observed singular bioaccumulation patterns of PFAS in different marine biota, identifying clear trends in the relative abundance of PFUnDA, PFHpA, PFNA and PFOA across species such as limpets, sea snails, mussels, sea urchins and starfish. However, it is important to acknowledge that this study does not fully address the potential uncertainties or variability in bioaccumulation rates both among different species and within populations of the same species. To account for a bit of this, we employed representative sampling across multiple locations and times and included a diverse range of species. Chemical analysis techniques were used to accurately quantify PFAS concentrations. Additionally, we measured environmental conditions and PFAS concentrations in water and sediment to understand their influence on bioaccumulation.

These chemicals are not naturally occurring in the marine environment, which raises concerns about their presence and impact. It should be noted that numerous other pharmaceuticals, herbicides, industrial chemicals and other endocrine-disrupting chemicals are also present in sewage effluents (Swartz et al. 2018b) and were found to be bioaccumulating in the same environment (Ojemaye and Petrik 2022). Moreover, certain PFASs, such as PFNA, are excessively persistent and remain unaffected by environmental biodegradation processes making them a concern for both the environment and human health due to their toxicity related to reproduction and growth (Martin et al. 2002; Stock et al. 2004; Butenhoff et al. 2004; Olsen et al. 2005; Taniyasu et al. 2005). Recently, PFASs such as PFOA have been banned, and a restriction proposal is under consideration by the European Chemical Agency because of their extreme persistence in the environment. The presence and impact of these compounds in the ecosystem raise significant concerns for both the environment and human health (Martin et al. 2002; Stock et al. 2004; Taniyasu et al. 2005). The exact causes for the elevated levels of PFAS at Simon’s Town (site 2) could be due to activities at the naval base located in that area, which subsequently find their way into the nearby wastewater treatment plants (WWTPs) or storm water drains. Additionally, their presence could be attributed to their use during naval training or in the maintenance and repair of ships. Moreover, the elevated levels of PFAS observed in False Bay may also be attributed to fire training exercises that utilize firefighting foams containing PFAS (Viberg and Eriksson 2017) as well as their use in the production of various household products in this region. These compounds are persistent and bioaccumulative in marine environments, making bioaccumulation an important contamination indicator in water-dwelling organisms (Mao et al. 2012). In general, this study highlights the significance of bioaccumulation as a crucial indicator of contamination in marine organisms.

PFNA emerged as the predominant PFAS across all sites, consistently exhibiting the highest concentration. The factors contributing to its elevated levels have been extensively discussed in earlier sections, encompassing the discharge volume of sewage, extensive use of PFNA in consumer products and its notable persistence in the environment. The urban surrounds of False Bay have been undergoing rapid urbanization and industrialization, contributing to the accumulation of PFAS in the biota.

### Seaweeds

**Fig. 6:**
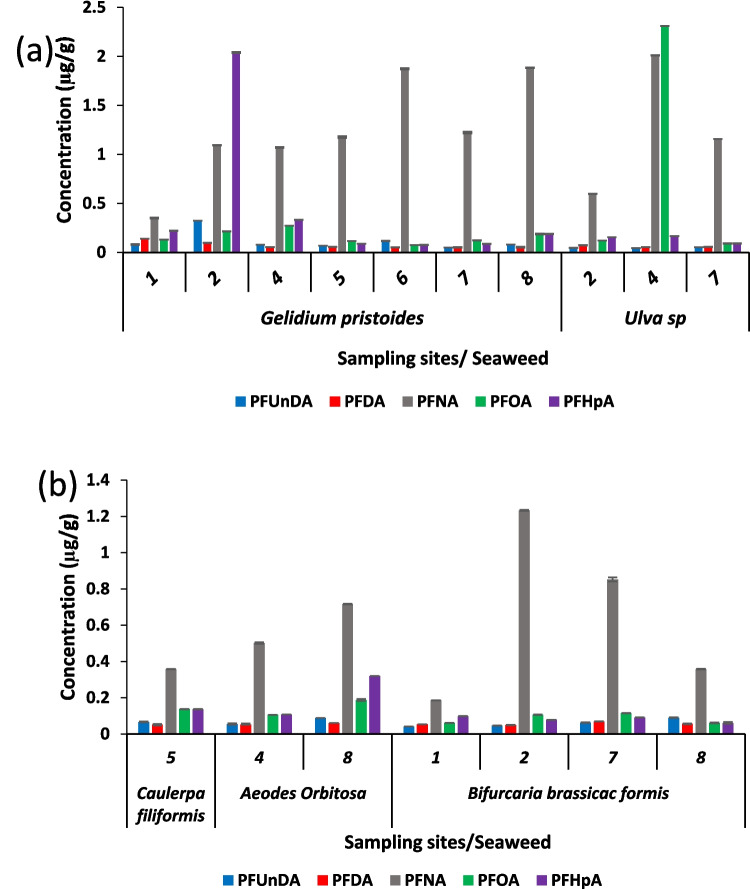
PFAS concentration in seaweed found in different sites

Seaweeds are considered to be one of the most appropriate organisms for monitoring contamination due to their abundance in the near shore coastal environment and their capacity to accumulate pollutants in marine environment (Anastasakis et al. 2011). The species of seaweed examined in this study included red algae, sea lettuce, hanging wrack, strap Caulerpa and slippery orbit whose abundance varied among the sites. The concentration of PFAS in the different seaweed species is shown in Fig. 6.

Red algae are seaweed that falls under the *Gelidiaceae* family. The gelling agents, frequently utilized in the manufacturing of various products, are derived from the agar extracts obtained from this particular seaweed including those used in the manufacturing of sweet and jelly food items, as well as in bacteriological culture bases (Bolton and Stegenga 2002; Deng et al. 2022). The seaweed was sampled from all the different sites in False Bay, apart from site 3. The detected levels of PFAS in red algae exhibited the following range: PFUnDA (0.0509–0.3225 µg/g dw), PFDA (0.0139–0.0971 µg/g dw), PFNA (0.3513–1.8839 µg/g dw), PFOA (0.1155–0.2719 µg/g dw) and PFHpA (0.0871–2.0400 µg/g dw). Notably, a significant level of PFHpA was detected in the red algae samples at site 2. Moreover, this species exhibited PFNA as the predominant PFAS, aligning with the study’s findings of elevated PFNA levels observed across all sites in other matrices.

Sea lettuce, also known as the green alga (*Ulva* sp.), which is a member of the *Ulvaceae* family, was specifically identified and only found at sites 2, 4 and 7 within False Bay. The concentrations of PFAS detected in *Ulva* sp. exhibited the following ranges: PFUnDA (0.0432–0.0514 µg/g dw), PFDA (0.0546–0.0728 µg/g dw), PFNA (0.5978–2.0092 µg/g dw), PFOA (0.0904–2.3092 µg/g dw) and PFHpA (0.0900–0.1666 µg/g dw). Considering that seaweed is used for medicinal and food purposes, the high levels of contamination raise concerns about the associated health risks, such as increased cancer risk, decreased fertility and immune system effects (Starling et al. 2017; Olsen et al. 2017). In 2018, the European Food Safety Authority (EFSA) conducted a reassessment of the health risks associated with the presence of PFOA in food.

The revised evaluation yielded significantly lower thresholds compared to previous assessments (Knutsen et al. 2018; European Food safety Authority 2020). The tolerable weekly intake (TWI) value published by EFSA in 2018 was 6 ng/kg body weight (BW) for PFOA, well below the corresponding value established by the Working Groups on Soil Protection (LABO) and on Water Issues (LAWA). Subsequently, in 2020, EFSA revisited its assessment, recommending a new TWI for the combined sum of four compounds PFOA, PFOS, PFHxS and PFNA at 4.4 ng/kg BW. This represents a further tightening of EFSA’s recommendation. Notably, this assessment marked a departure as it considered the combined toxicity of contaminant groups, taking into account cocktail effects for the first time (Federal Ministry for Nature Conservation Nuclear Safety and Consumer Protection 2022).

This shows that even for mixture of contaminants the levels need to be at a very low or no concentration, which in the case of this study is higher.

Strap Caulerpa is a bright green seaweed that often forms grass-like beds in sandy gulleys. It belongs to the family *Caulerpacea* and was only found at site 5. The underlying reasons for the elevated presence of PFNA in this particular seaweed species have been previously discussed.

The seaweed species, slippery orbits, belonging to the family *Halymeniaceae*, was only found at sites 4 and 8. PFASs were detected in samples from both sites; the concentration of these compounds, including PFNA, was found to be higher in slippery orbits samples from site 8 compared to site 4, consistent with observations in other samples. The levels of PFAS identified in the slippery orbits samples from both sites are similar to the concentrations found in other seaweed species.

The species Hanging wrack belongs, a type of brown algae seaweed, is commonly used in various food dishes. It was collected from four sites (1, 2, 3 and 8) The concentrations of different PFAS in hanging wrack ranged from 0.0409 to 1.2330 µg/g dw (Fig. 6).

Notably, all seaweed samples contained these PFASs, highlighting the extensive release of these chemicals in the ecosystem of False Bay. Among these compounds, PFNA displayed the highest concentration across all sites, which aligns with our findings in other biota samples. The elevated levels of PFNA can be attributed to its resistance to biodegradation, widespread use in consumer products and discharge into the marine environment through effluent. The accumulation of PFAS observed in various seaweeds species varied significantly, potentially due to their different patterns of uptake and metabolism of PFAS chemicals and their fluctuating availability in seawater and sediment. The presence of these PFAS in seaweed samples indicates that although these chemicals are present in the surrounding media at low concentrations, they are accumulating in the seaweed samples. The study indicates that PFASs are accumulating in all the seaweed samples, with PFNA being the most prevalent compound.

In literature, contamination in marine organisms (for instance bivalves and fish) has received more attention, but the pollution and associated harmful effects on seaweeds are generally neglected. This study demonstrates that the contamination levels in seaweeds can indeed be compared to those observed in other marine invertebrates in False Bay. As the use of seaweeds for medicinal and food continues to grow (Chen et al. 2018), it is crucial to recognize the potential harm that contaminated seaweed may cause to consumers (humans and marine organisms). Therefore, it is important to conduct monitoring and estimate the health risk posed by emerging contaminants in seaweeds.

By comparison, the concentration of PFAS in bivalves and invertebrates (mussels, clams, starfish, gastropod, snail) from different locations ranged from nd to 120.75 ng/g in Italy (Chiesa et al. 2018); 0.11–1.1 ng/g in the Ariake Sea, Japan (Kobayashi et al. 2018); nd–1062 ng/g in the harbour areas in Spain (Zabaleta et al. 2014); < DL–10.8 ng/g along the west coast of Korea (Naile et al. 2013; Hong et al. 2015); nd–0.22 ng/g from Taihu China (Xu et al. 2014); nd–2.26 ng/g from Northern Bohai Sea (Wang et al. 2011) and 0.06–0.04 ng/g in Vietnam (Lam et al. 2017). It was observed that the concentrations of PFAS were either lower or higher than the concentration found in this study. Similar concentrations of PFAS were also reported in the biota samples from Jiangsu, China (Wu et al. 2012); China (Zhang et al. 2011; Chiesa et al. 2018); Bohai Sea, north-eastern China (Guo et al. 2019) and Catalonia, Spain (Domingo et al. 2012).

However, it is likely that the disparities in the levels of PFAS between this study and the studies mentioned previously are due to location-specific differences in PFAS, distinct release patterns from originating sources that contribute to variations in the resulting water concentrations as well as prevailing winds and currents and differences in the age and species type of the biota. The varying pollution profiles observed between the different sites suggest that PFAS levels differ across geographic regions globally but are ever present. Nevertheless, the results of this study generally indicate significantly higher concentrations of PFAS in various biota and seaweeds in False Bay than those reported in some of the cited studies. This underscores the urgent need to limit the release of effluent containing these compounds into the coastal/marine milieu to prevent serious consequences of their side effects on both marine organisms and humans who consume seafood. Also, analysing the chemical composition of PFAS in sewage is a more effective and practical method for tracking and assessing the dispersion of sewage pollution in the marine environment compared to using *Escherichia coli* as an indicator for short-term dispersion modelling as is the practices of the city as noted in the CSIR report (CSIR 2017). Additionally, strict measures need to be put in place to ban or prevent the sale of these toxic contaminants as their tendency is to move up the food chain and bioaccumulate. Ultimately, the public should be educated on the importance of continuously protecting the marine environment from hazardous chemical compounds by adopting lifestyle modifications to mitigate marine environmental pollution.

### Risk assessment

#### Environmental risks of individual compound

The environmental risk was calculated for individual compounds in seawater, and the results are presented in Table 3. The values of toxic units (TUs = MEC/EC50) for the three trophic levels (algae, invertebrate and fish) in the various seawater samples taken from the eight sites did not exceed the critical threshold of 10^−3^ set for tier 1 as mandated by the guideline of FDA for compounds.

**Table 3:** Toxic unit calculated for individual PFAS

Site number	Trophic level	PFHpA	PFOA	PFNA	PFDA	PFUnDA
	Algae	6.7E − 09	1.77E − 07	1.88E − 07	1.65E − 07	7.34E − 09
1	Invertebrate	1.27E − 07	1.1E − 06	2.02E − 07	2.27E − 08	4.15E − 08
	Fish	0	1.43E − 06	1.56E − 07	5.47E − 08	6.91E − 08
	Algae	4.66E − 09	6.76E − 08	8.03E − 08	2.31E − 07	9.7E − 09
2	Invertebrate	8.84E − 08	4.19E − 07	8.65E − 08	3.18E − 08	5.48E − 08
	Fish	0	5.46E − 07	6.66E − 08	6.81E − 08	9.13E − 08
	Algae	1.71E − 09	3.22E − 08	1.11E − 07	1.16E − 07	1.42E − 08
3	Invertebrate	3.25E − 08	2E − 07	1.19E − 07	1.6E − 08	8.05E − 08
	Fish	0	2.61E − 07	9.16E − 08	3.84E − 08	1.34E − 07
	Algae	6.37E − 09	1.23E − 07	3.54E − 08	2.42E − 07	2.21E − 08
4	Invertebrate	1.21E − 07	7.66E − 07	3.81E − 08	3.33E − 08	1.25E − 07
	Fish	0	9.98E − 07	2.93E − 08	8.03E − 08	2.08E − 07
	Algae	5.35E − 09	9.77E − 08	6.41E − 08	2.21E − 07	1.04E − 08
5	Invertebrate	1.01E − 07	6.06E − 07	6.91E − 08	3.04E − 08	5.87E − 08
	Fish	0	7.9E − 07	5.31E − 08	7.31E − 08	9.78E − 08
	Algae	3.64E − 09	4.76E − 08	4.38E − 08	3.24E − 07	9.79E − 09
6	Invertebrate	6.91E − 08	2.95E − 07	4.72E − 08	4.45E − 08	5.53E − 08
	Fish	0	3.85E − 07	3.63E − 08	1.07E − 07	9.22E − 08
	Algae	3.67E − 09	4.7E − 08	5.51E − 08	7.03E − 07	9.41E − 09
7	Invertebrate	6.96E − 08	2.92E − 07	5.94E − 08	9.66E − 08	5.32E − 08
	Fish	0	3.8E − 07	4.57E − 08	2.33E − 07	8.87E − 08
	Algae	4.24E − 09	6.13E − 08	4.38E − 08	2.29E − 07	9.98E − 09
8	Invertebrate	8.05E − 08	3.81E − 07	4.72E − 08	3.15E − 08	5.64E − 08
	Fish	0.00E + 00	4.96E − 07	3.63E − 08	7.59E − 08	9.4E − 08

#### Environmental risks assessment of chemical mixtures

Risk assessment for the total PFAS compound mixture was also evaluated in the seawater. Table 4 reports the STUs evaluated for each trophic level (algae, invertebrate and fish) at each site and the RQs based on the MEC/PNEC ratios, and the STUs (RQ_(STU)_). In general, the RQs evaluated using the two approaches were not different. Table 4 shows the STU range from 2.75E − 07 (algae, site 3) to 1.71E − 06 (fish site 1).

**Table 4:** Summary of mixture toxicity assessments

		1	2	3	4	5	6	7	8
Max TU	Algae	1.88E − 07	2.31E − 07	1.16E − 07	2.42E − 07	2.21E − 07	3.24E − 07	7.028E − 07	2.3E − 07
	invertebrate	1.1E − 06	4.19E − 07	2E − 07	7.66E − 07	6.06E − 07	2.95E − 07	2.916E − 07	3.8E − 07
	Fish	1.43E − 06	5.46E − 07	2.61E − 07	9.98E − 07	7.9E − 07	3.85E − 07	3.798E − 07	5E − 07
STU	Algae	5.44E − 07	3.93E − 07	2.75E − 07	4.3E − 07	3.98E − 07	4.28E − 07	8.18E − 07	3.5E − 07
	invertebrate	1.49E − 06	6.81E − 07	4.48E − 07	1.08E − 06	8.66E − 07	5.12E − 07	5.704E − 07	6E − 07
	Fish	1.71E − 06	7.72E − 07	5.25E − 07	1.32E − 06	1.01E − 06	6.21E − 07	7.47E − 07	7E − 07
RQ(MEC/PNEC)		0.0020	0.0010	0.0007	0.0016	0.0013	0.0009	0.0013	0.0009
RQ(STU)		0.0017	0.0008	0.0005	0.0013	0.0010	0.0006	0.0007	0.0007
RQ(MEC/PNEC)/RQ(STU)		1.17	1.35	1.26	1.22	1.26	1.48	1.74	1.35
Median	Algae	1.65E − 07	6.76E − 08	3.22E − 08	3.54E − 08	6.41E − 08	4.38E − 08	4.699E − 08	4.4E − 08
	invertebrate	1.27E − 07	8.65E − 08	8.05E − 08	1.21E − 07	6.91E − 08	5.53E − 08	6.96E − 08	5.6E − 08
	Fish	6.91E − 08	6.81E − 08	9.16E − 08	8.03E − 08	7.31E − 08	9.22E − 08	8.865E − 08	7.6E − 08
Sum of TU/median TU	Algae	3.29E + 00	5.82E + 00	8.53E + 00	1.21E + 01	6.21E + 00	9.78E + 00	1.74E + 01	7.96E + 00
	invertebrate	1.17E + 01	7.87E + 00	5.57E + 00	8.97E + 00	1.25E + 01	9.25E + 00	8.20E + 00	1.06E + 01
	Fish	2.47E + 01	1.13E + 01	5.73E + 00	1.64E + 01	1.39E + 01	6.73E + 00	8.43E + 00	9.24E + 00

RQ (MEC/PNEC) = ∑MEC/PNEC with PNEC = min (EC50 for all trophic level) *1000. RQ (STU) = max (∑MEC/EC50 for all trophic level) *1000

*Max TU* maximum toxic unit of an individual PFAS for the indicated trophic level and seawater, *STU* sum of toxic units, i.e., = ∑MEC/EC50 for each trophic level

In accordance with the methodology for establishing environmental quality standards outlined in the technical guidance document: Registration, Evaluation, Authorisation and Restriction of Chemicals (REACH) (ECHA 2008; EU Commission 2011), when assessing the environmental risk using acute toxicity data from three feeding levels, an assessment factor of 1000 is recommended; hence, the resulting RQ_STU_ values for the seawater ranged between 0.0006 and 0.02 show that there is no potential environmental risks associated with PFAS in the majority of these situations. Regardless of the notable differences in the concentration of the PFAS mixture in seawater at eight studied sites (Fig. 2), a positioning of the three examined feeding levels revealed that fish are an extremely sensitive group (Table 4), followed by invertebrates and algae, which were least sensitive (Fish > invertebrate > algae) in all eight analysed scenarios. While there were no individual compounds at the chosen trophic level that posed any risk, it is crucial to be concerned about the overall risk posed by the mixture of PFAS, as this study only considered five PFAS compounds, and it is expected that the RQs should be below 1. The presence of these compounds in the environment is thus consistently in the form of a mixture, which indicates that it is not only the studied compounds that will be present in the examined locations as there are many thousands of PFASs that are in use globally (Wang et al. 2017). It is important to note that the values of toxicity calculated using standard protocols in this study do not represent the overall toxicity of seawater. Rather, they specifically only assess the combined toxicity contributed by the tested PFAS incorporated in the analytical survey.

Noteworthy, considering that some PFAS compounds are identified as carcinogenic, it would indeed necessitate a higher safety factor or even a “zero tolerance” policy upon their detection. Incorporating higher safety factors can help account for the long-term and potentially irreversible health impacts of such substances. In such cases, even minimal exposure levels can be unacceptable; hence, a “zero tolerance” approach might be advocated to ensure complete protection of public health and the environment. Incorporating an EC10 (the concentration at which 10% of the population experiences a response) approach could provide further ecotoxicological insights. EC10 is a more sensitive measure and can help identify potential risks at lower concentrations, which is crucial for understanding sub-lethal effects that might not be apparent with EC50.

## Conclusion

The False Bay marine environment is impacted by chemically contaminated effluent from seven different WWTPs, and from very intense industrial, naval and harbouring activities. The PFASs detected in sewage at one of the WWTP discharging effluent into the marine environment were used to trace the chemical fingerprint of sewage through monitoring PFAS levels at various locations in the bay. To assess the contamination levels in False Bay, a comprehensive analysis was conducted on seawater, sediment and biota samples collected at selected locations to determine the ranges of five PFAS at eight different sites. Fluctuating and relatively low levels of PFAS were detected in the effluent released from the WWTP, but the constant discharge of partially treated effluents from WWTPs into the bay impacted the receiving marine environment considerably. The study focused only on specific PFAS; however, there are numerous unknown and undetected PFAS. The study demonstrates that primary and secondary treatments of sewage effluents are not adequate to protect the receiving environment. The results of monitoring of PFAS as a tracer of the chemical fingerprint of sewage showed the extensive dispersion and impact zone of sewage effluents upon the marine environment. Across all instances, the presence of PFAS in seawater was consistently detected at low ng/L levels, whereas the concentrations in spatially separated samples of biota and sediments were three orders of magnitude higher. Limpet samples were found to be the most suitable species to use as biomonitors of contamination due to their ubiquitous presence at all sites. The study also demonstrates that using the PFAS chemical fingerprint in sewage as tracer of sewage dispersion in the marine environment is more realistic than applying momentary plume dispersion modelling using *Escherichia coli* as indicator to determine impact zones of sewage pollution, for environmental impact assessment as demonstrated previously in the CSIR report. Bioaccumulation of these compounds was found in an impact zone that extended more than 60 km from point of discharge and was species-specific and dependent on the specific PFAS structure. PFNA was the dominant compound detected in all the biota samples. The study cautions that the risk quotients of the selected PFAS are only indicative, as the five selected PFAS compounds are merely representative, as numerous analogues of these compounds may also simultaneously be accumulating in the biota. It is crucial to be concerned about the cumulative risk posed by the mixture of diverse PFAS as well as other co-occurring contaminants. Given the significant persistence, bioconcentration, bioaccumulation and biomagnification potential of these chemical compounds, there is a concern that continuous release of toxic effluent into the sea may have acute or long-term effects on marine biota that are not fully investigated. To mitigate this risk, adequate treatment of wastewaters beyond primary and secondary level is needed before their discharge into the environment. Moreover, the manufacture and sale of commodities carrying PFAS should be screened and limited to essential applications and a substituting process. South Africa may also take the lead in the phase out of the production and importation of persistent PFAS compounds, in accordance with the Stockholm convention. These actions are crucial in reducing the contamination of the marine and terrestrial ecosystem and to safeguard ecosystem integrity and the general public.

## Supplementary Information

Below is the link to the electronic supplementary material.

Supplementary file1 (DOCX 37 KB)

## Data Availability

The authors declare that the data supporting the findings of this study are available within the paper and its Supplementary Information files. Should any raw data files be needed in another format they are available from the corresponding author upon reasonable request. Source data are provided with this paper.
