# Supplementary material for: The efficacy of wastewater treatment plant on removal of perfluoroalkyl substances and their impacts on the coastal environment of False Bay, South Africa
Source: Environ Sci Pollut Res Int. 2024 Nov 18;31(56):64772–95. doi: 10.1007/s11356-024-35509-7 (PMC11624228; doi:10.1007/s11356-024-35509-7)
Supplement: Supplementary file 1 — Supplementary file1 (DOCX 37 KB) [file 11356_2024_35509_MOESM1_ESM.docx]

**The efficacy of wastewater treatment plant on removal of perfluoroalkyl substances and their impacts on the coastal environment of False Bay, South Africa**

Cecilia Y. Ojemaye*^12^, Adeola Abegunde^1^, Lesley Green^2^ Leslie and Petrik^1^

^1^Environmental and Nano Science Group, Department of Chemistry, University of the Western Cape, Cape Town, South Africa

^2^Environmental Humanities South and Department of Anthropology, University of Cape Town, Cape Town, South Africa

Correspondence: [cecilia.ojemaye@uct.ac.za](mailto:cecilia.ojemaye@uct.ac.za)

1. **Sample preparation**
   1. **Water samples**

Seawater samples of 500 mL volume were used for analysis. The pH of the previously filtered seawater samples was adjusted to 6 with a few drops of 0.1M of HCl or NaOH. With the aid of a vacuum pump and manifold, SPE was carried out using the HLB cartridges preconditioned with methanol (7 mL), followed by Milli-Q water (7 mL). Each of the 500 mL filtered seawater samples was separately loaded into their respective preconditioned cartridges, using 5 mL/min as the flow rate. The cartridges were left to air-dry under vacuum for 20 min, and 15 mL of methanol was used to elute the analytes using a flow rate of 1 mL/min. Subsequently, the eluate was concentrated to 2 mL using a nitrogen gas stream. Internal standards (50 µL, of a 50 ng/mL solution) was added to each sample. further centrifuged for 25 min, and transferred into liquid chromatography (LC) vials after filtration using a polyvinylidene fluoride syringe filter (particle retention 0.2 μm, GF/C diameter 13 mm) prior to analysis on LC–MS (Petrik et al., 2017)(Ojemaye & Petrik, 2021).

- 1. **Biota and sediment samples**

Tissue from the respective marine organisms was freeze dried and ground into a fine powder, while sediment samples were air-dried. Approximately 10 g was weighed and placed into an extraction thimble. The sample was extracted with 100 mL methanol/ acetone 3:1 (v/v) using a soxhlet extraction method. The extract was concentrated to 10 mL using a rotary evaporator at reduced pressure, and the sample pH was adjusted to 6 by adding 1 M NaOH or HCl so as to allow the precipitation of lipids. The extract was centrifuged at 3000 rpm for 20 min. The supernatant was transferred to glass/polypropylene bottles and Millipore water was added to make up to a volume of 100 mL. The SPE procedure was the same as that used to treat seawater samples using 1 mL/min as the flow rate. A mixture of internal standards (50 μL, 50 ng/mL) was added to the final extract and analyzed using LC-mass spectrometry (MS). Recovery standards were added to each sample prior to analysis (Ojemaye & Petrik, 2021).

- 1. **Chromatographic conditions**

The chromatographic separations were performed with the Acquity UPLC^TM^ instrument (Waters, Milford, MA, USA). Simultaneous determination of all the compounds of interest was achieved using an Acquity UPLC BEH C18 column 2.1 mm × 100 mm, 1.7-μm with an Acquity BEH C18 1.7-μm VanGuardTM precolumn (2.1 mm × 5 mm), supplied by Waters. The column temperature was set to 60 °C. The mobile phase consisted of a mixture of 2mM ammonium acetate (solvent A) in water, and 2mM ammonium acetate in methanol (solvent B). Linear gradient elution of 0.35 mL/min was used, starting with a mixture of 90% solvent A and 10% solvent B for 8 min. At 9 min, the percentage of both solvents were adjusted to 10% of solvent A and 90% of solvent B. At 10 min the percentage of solvent B was increase to 100%, at 10 mins 10secs, the percentage was maintained at 90% of solvent A and 10% of solvent B. A volume of 5 μL of each sample was injected into the LC/MS system. Standards and the test samples were subjected to a 12-min chromatographic run.

- 1. **Mass spectrometry**

The UPLC was coupled to a triple quadrupole mass spectrometer (Xevo TQ-MS), with an electrospray ionisation source. During optimisation, a multiple reaction monitoring scan mode was generated for all analytes. In addition, for maximum sensitivity, other conditions such as source temperature, capillary voltage, cone voltage, cone gas flows and desolution temperatures were standardised. This standardisation was achieved by direct injection of stock solutions with a concentration of 10 μg/mL. A capillary voltage of 3.5 kV, desolvation gas (N_2_) flow of 800 L/h, source temperature of 140 °C and desolvation temperature of 400 °C were finally used. The analytical operation control and data processing were performed with Masslynx software.

- 1. **Precision**

Four sets of quality control (QC) samples, each consisting of six replicates, were meticulously prepared and subjected to analysis over the course of three separate days. The concentration of perfluorinated compounds (PFCs) in the QC samples was computed using the linear regression equation derived from the calibration curve generated from calibrators run within the same batch. The precision within a single day for the QC samples consistently measured at 11.5% or lower for all QC levels across the three validation days. Furthermore, the precision across different days for all four QC levels remained consistently below 14.8%. The precision criteria for validation acceptance dictate a relative standard deviation (RSD) within ±15% for each QC sample level, except for the lower limit of quantification (LOQ), where it should not exceed ±20%. Importantly, all results for method precision comfortably met the criteria for validation data acceptance

Table 1: Acute toxicity data for available contaminants across the three trophic levels

|  | Algae | | | Invertebrate | | | Fish | | |
| --- | --- | --- | --- | --- | --- | --- | --- | --- | --- |
| Compounds | EC_50_  (mg L^–1^) | Species | Reference | EC_50_  (mg L^–1^) | Species | Reference | EC_50_/LC_50_  (mg L^–1^) | Species | Reference |
| PFHpA | 1896.75 | *C. vulgaris* | (Latała et al., 2009) | >100 | *D. magna* | (Hoke et al., 2012) | - | - | - |
| PFOA | 96.2 | *P. subcapitata* | (Rosal et al., 2010) | 15.50 | *P lividus* | (Mhadhbi et al., 2012) | 11.90 | *P maxima* | (Mhadhbi et al., 2012) |
| PFNA | >100 | *P. subcapitata* | (Hoke et al., 2012) | 92.80 | *D. magna* | (Durjava et al., 2012) | 120.64 | - | (Durjava et al., 2012) |
| PFDA | 10.6 | *P. subcapitata* | (Hoke et al., 2012) | 77.10 | *D. magna* | (Hoke et al., 2012) | 32.0 | *O. mykiss* | (Hoke et al., 2012) |
| PFUnDA | 318.66 | *P. subcapitata* | (Durjava et al., 2012) | 56.40 | *D. magna* | (Durjava et al., 2012) | 33.840 | - | (Durjava et al., 2012) |

**Table 2: Assessment factors proposed by the European Chemicals Agency for deriving PNEC_water_ for saltwater for different data sets**

| **Data set** | **Assessment factor** |
| --- | --- |
| Lowest short-term L(E)C50 from representatives of three taxonomic groups (algae, crustaceans and fish) of three trophic levels | 10,000 |
| At least one short-term L(E)C50 from each of three  trophic levels of the base set (fish, Daphnia and algae | 1,000 |
| Two long-term NOECs from species representing two trophic levels (algae and/or crustaceans and/or fish) | 500 |
| One long-term NOEC (either fish or Daphnia) | 100 |
| Two long-term NOECs from species representing two  trophic levels (fish and/or Daphnia and/or algae) | 50 |
| Long-term NOECs from at least three species (normally  fish, Daphnia and algae) representing three trophic levels | 10 |

**References**

Delamore, P. (2018). *Auckland Tauranga Wellington Christchurch Impact of Per and Poly Fluoroalkyl Substances on Ecosystems Ministry for the Environment solutions for your environment*. http://www.pdp.co.nz

Ding, G.-H., Frömel, T., van den Brandhof, E.-J., Baerselman, R., & Peijnenburg, W. J. G. M. (2012). Acute toxicity of poly- and perfluorinated compounds to two cladocerans, Daphnia magna and Chydorus sphaericus. *Environmental Toxicology and Chemistry*, *31*(3), 605–610. https://doi.org/10.1002/etc.1713

Ding, G., Wouterse, M., Baerselman, R., & Peijnenburg, W. J. G. M. (2012). Toxicity of polyfluorinated and perfluorinated compounds to lettuce (Lactuca sativa) and green algae (Pseudokirchneriella subcapitata). *Archives of Environmental Contamination and Toxicology*, *62*(1), 49–55. https://doi.org/10.1007/s00244-011-9684-9

Durjava, M., Kolar, B., & Peijnenburg, W. (2012). *Case studies on the Development and Application of in-Silico Techniques for Environmental hazard and Risk assessment. National Institute of Public Health and the Environment (RIVM), Laboratory for Ecological Risk Assessment*.

Hoke, R. A., Bouchelle, L. D., Ferrell, B. D., & Buck, R. C. (2012). Comparative acute freshwater hazard assessment and preliminary PNEC development for eight fluorinated acids. *Chemosphere*, *87*(7), 725–733. https://doi.org/10.1016/J.CHEMOSPHERE.2011.12.066

Latała, A., Nedzi, M., & Stepnowski, P. (2009). Acute toxicity assessment of perfluorinated carboxylic acids towards the Baltic microalgae. *Environmental Toxicology and Pharmacology*, *28*(2), 167–171. https://doi.org/10.1016/j.etap.2009.03.010

Lu, G. H., Liu, J. C., Sun, L. S., & Yuan, L. J. (2015). Toxicity of perfluorononanoic acid and perfluorooctane sulfonate to Daphnia magna. *Water Science and Engineering*, *8*(1), 40–48. https://doi.org/10.1016/j.wse.2015.01.001

Mhadhbi, L., Rial, D., Pérez, S., & Beiras, R. (2012). Ecological risk assessment of perfluorooctanoic acid (PFOA) and perfluorooctanesulfonic acid (PFOS) in marine environment using Isochrysis galbana, Paracentrotus lividus, Siriella armata and Psetta maxima. *Journal of Environmental Monitoring*, *14*(5), 1375–1382. https://doi.org/10.1039/c2em30037k

Ojemaye, C. Y., & Petrik, L. (2021). Pharmaceuticals and Personal Care Products in the Marine Environment Around False Bay, Cape Town, South Africa: Occurrence and Risk-Assessment Study. *Environmental Toxicology and Chemistry*, *00*(00), 1–21. https://doi.org/10.1002/etc.5053

Petrik, L., Green, L., Abegunde, A. P., Zackon, M., Sanusi, C. Y., & Barnes, J. (2017). Desalination and seawater quality at Green Point, Cape Town: A study on the effects of marine sewage outfalls. *South African Journal of Science*, *113*(11/12), 1–10. https://doi.org/10.17159/sajs.2017/a0244

Rosal, R., Rodea-Palomares, I., Boltes, K., Fernández-Piñas, F., Leganés, F., & Petre, A. (2010). Ecotoxicological assessment of surfactants in the aquatic environment: Combined toxicity of docusate sodium with chlorinated pollutants. *Chemosphere*, *81*(2), 288–293. https://doi.org/10.1016/J.CHEMOSPHERE.2010.05.050

Verbruggen, E., Wassenaar, P., & Smit, C. (2002). Water quality standards for Alabama. In *Federal Register* (Vol. 67, Issue 205, pp. 65255–65270).
